# Accounting for uncertainty when assessing association between copy number and disease: a latent class model

**DOI:** 10.1186/1471-2105-10-172

**Published:** 2009-06-06

**Authors:** Juan R González, Isaac Subirana, Geòrgia Escaramís, Solymar Peraza, Alejandro Cáceres, Xavier Estivill, Lluís Armengol

**Affiliations:** 1 Center for research in environmental epidemiology (CREAL), Barcelona, Spain; 2 CIBER en Epidemiología y Salud Pública (CIBERESP), Barcelona, Spain; 3 Institut Municipal d'Investigació Mèdica (IMIM), Barcelona, Spain; 4 Genes and Disease Program, Center for Genomic Regulation, Barcelona, Spain

## Abstract

**Background:**

Copy number variations (CNVs) may play an important role in disease risk by altering dosage of genes and other regulatory elements, which may have functional and, ultimately, phenotypic consequences. Therefore, determining whether a CNV is associated or not with a given disease might be relevant in understanding the genesis and progression of human diseases. Current stage technology give CNV probe signal from which copy number status is inferred. Incorporating uncertainty of CNV calling in the statistical analysis is therefore a highly important aspect. In this paper, we present a framework for assessing association between CNVs and disease in case-control studies where uncertainty is taken into account. We also indicate how to use the model to analyze continuous traits and adjust for confounding covariates.

**Results:**

Through simulation studies, we show that our method outperforms other simple methods based on inferring the underlying CNV and assessing association using regular tests that do not propagate call uncertainty. We apply the method to a real data set in a controlled MLPA experiment showing good results. The methodology is also extended to illustrate how to analyze aCGH data.

**Conclusion:**

We demonstrate that our method is robust and achieves maximal theoretical power since it accommodates uncertainty when copy number status are inferred. We have made R functions freely available.

## Background

With the recent technological advances, various genome-wide studies have uncovered an unprecedented number of structural variants throughout the human genome [1-3], mainly in the form of copy number variations (CNVs). The considerable number of genes and other regulatory elements that fall within these variable regions make CNVs very likely to have functional and, ultimately, phenotypic consequences [4,5]. In fact, recent studies have reported a correlation between copy number of specific genes and degree of disease predisposition [6-8], indicating that identification of DNA copy number is important in understanding genesis and progression of human diseases.

Several techniques and platforms have been developed for genome-wide analysis of DNA copy number, such as array-based comparative genomic hybridization (aCGH). The goal of this approach is to identify contiguous DNA segments where copy number changes are present. The ability of aCGH to distinguish between different numbers of copies is limited, so various quantitative techniques are required for more precise, targeted analysis of genomic regions. For known CNVs, real time PCR assays can be used to compare the copy number status of particular loci in cases and controls. Individuals are typically binned into copy number categories using pre-defined thresholds of probe signal intensity. Recently, Multiplex Ligation-dependent Probe Amplification (MLPA) [9] has also been used to quantify copy number classes. This method allows the analysis of several loci at the same time in a single assay. MLPA is usually used to identify gains or losses in test samples with respect to controls [10], but it can also be used in the context of association studies in a case-control or cohort settings [11,12].

The statistical methods used in CNV-disease association studies are currently very simple. Quantitative methods give CNV probe signal intensity measurements for each individual as a continuous variable, from which copy number status is inferred, generally using pre-defined thresholds. Differences in copy number distribution between cases and controls are then assessed using *χ*^2^, Fisher or Mann-Whitney tests [6,13,14]. However, the distribution of CNV probe measurements is continuous and multimodal, meaning that signal intensity should be considered as a mixture of curves. In many instances, these curves overlap with various underlying distributions leading to uncertainty. Therefore, scoring copy number by binning and then assessing the association may lead to misclassification and unreliable results.

Ionita-Laza et al. (2009) pointed out that it is not inmediately clear how this uncertainty of CNV calling should be incorporated in the statistical analysis [15]. To overcome this difficulty in assessing association between CNVs and disease, we propose a latent class (LC) model that incorporates possible uncertainty that appear when CNV calling is performed. After inferring copy number using Gaussian finite mixture distributions, or any other calling algorithm, the model assesses the relationship between the trait and a CNV using a mixture of generalized linear models. Association is then tested using a likelihood ratio procedure. We validate and compare our method with existing methods through a simulation study. We then illustrate how to test association between CNVs and the trait by using two real examples. One of them corresponds to a case-control study using data from a MLPA experiment where the true copy number status is known. The second example belongs to a study where breast cancer cell lines are analyzed using aCGH.

## Methods

### Inference of copy number status

**Figure 1:**
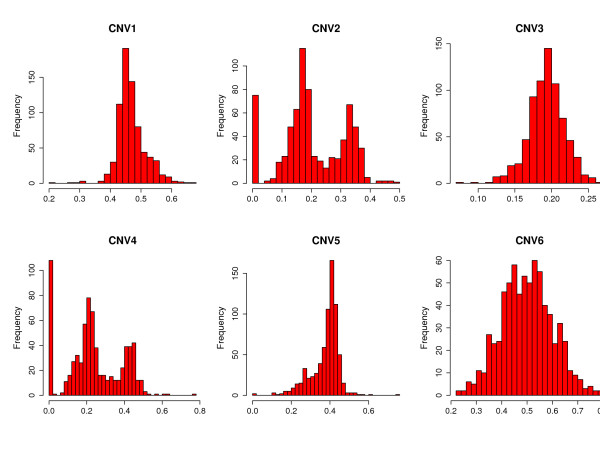
**CNV quantitative measurements**. Examples of CNV data showing different clustering quality and copy number status.

Let us assume that we observe *I *individuals from a given population, consisting of  mutually exclusive latent classes *c *= 1, ...,  (e.g. copy number status). Instead of observing these classes, we observe a surrogate variable, *X*, corresponding to a continuous variable arising from any quantitative method. For instance, in targeted studies using MLPA or real-time PCR, *X *corresponds to peak intensities for each CNV probe. In the context of a whole genome scan, one may have quantitative data from aCGH or any other platform such as Illumina or Affymetrix, where, for each probe, the variable *X *corresponds to a ratio of intensities. Figure 1 shows a number of possible distributions that signal intensities may have. Some variants clearly show different underlying copy number status with multimodal signal intensities distributions (CNV2, CNV4 and CNV6). In other cases, where the existence of different copy numbers is not clear, inferring copy number by binning the data may be difficult or unfeasible.

For each CNV variant, we are interested in classifying the subjects into the  classes using the surrogate variable *X*. We propose to model the unobserved latent classes using a finite mixture model with  components of the form

(1)

where *N*(·|*η*_*c*_, ) is the Gaussian distribution with **Θ **denoting all model parameters (e.g., **Θ **= (*η*_*c*_, ), *c *= 1, ..., ), and *x *is the surrogate variable that corresponds to the quantitative measure of copy number status. For the component weights *π*_*c *_it holds that



The value of  to be used is chosen by applying Bayesian Information Criteria (BIC) [16]. This mixture model approach for calling is similar to some used for the analysis of aCGH data [17,18] where correlation among probes should be considered. When analyzing MLPA data, it should be pointed out that in some instances, especially when there are individuals with 0 copies, the intensity distributions (see CNV2 and CNV4 in Figure 1) for a null allele is meant to be equal to 0. However, due to experimental noise it is fact that in some cases this ratio shows values that slightly deviate from this theoretical value. After our experience with hundreds of home-made MLPA probes, the value for null alleles is typically below 0.1; nevertheless, we recommend this parameter to be determined experimentally for each of the probes used in the MLPA experiments using the appropriate control samples. For these cases, the procedure used to estimate the parameters in (1) fails because the underlying distribution of individuals with 0 copies is not normal. In these situations we propose to fit the following mixture model to determine the latent classes

(2)

where *τ *is given by the user, as previously indicated, , ℐ denotes an indicator function, and



The posterior probabilities are used to segment data by assigning each individual to a given copy number status corresponding to the class with maximum posterior probability (MAP). After fitting this finite mixture model, we can perform a goodness-of-fit test using *χ*^2 ^test statistic. Finite mixture parameters can be estimated using the EM algorithm [19,20] or Newton-type procedures [20]. Then, the posterior probability that individual *i *with an observed value *x *belongs to copy number class *j *is given by

(3)

### Latent class model

#### Discrete traits

Let us suppose that copy number status is associated with a binary phenotype (case-control). The association is typically assessed using a *χ*^2 ^test for the contingency table (Table 1). Misclassification in the table (due to uncertainty when inferring CNVs) is incorporated when we assign each individual to a given class *c *using *maximum a-posteriori probability *(MAP). Thus, this problem can be seen as an association study with misclas-sification ("measurement error") [21]. It is well known that misclassification of covariates has important implications for parameter estimates and statistical inference [22]. Some approaches account for such error [23,24]. These are, however, based on performing validation studies in a subsample. In the present context, this is unfeasible because hundreds of genes are normally analyzed at a time, and the technology may have a different sensitivity and specificity for each of the inspected loci. Therefore, we propose to use the posterior probability of belonging to each latent class to model the degree of misclassification of copy number status. We then take this information into account in the association model.

**Table 1:** Contingency table of disease status and copy number category

	Copy number status				
		
Disease	1	2	⋯		Total
Cases	*r*_1_	*r*_2_	⋯		R
Controls	*s*_1_	s_2_	⋯		S

Conditioning on cluster *c*, we have that

(4)

where ***β ***= (*β*_1_, ..., *β*_*c*_), *c *= 1, ...,  is our vector of parameters, and



Then, equation (4) can be rewritten as



Now, we consider that copy number status is measured with error (i.e., the latent class is not known). Therefore, we are modeling the probability of being an affected individual as a mixture of  binomial variables, as follows:



where *w*_*ic *_is the posterior probability that individual *i *belongs to copy number class *c*, given in (3). Therefore, assuming conditional independence of case-control status, given latent class, the likelihood function for model parameters ***β ***can be written as

(5)

We can then simply compute the odds ratio (OR) of belonging to class *c *with respect to a given reference *r *as

(6)

#### Quantitative traits

We now consider the case where our phenotype, *Y*, is continuous. We assume that *Y *|*c N*(*μ*_*c*_, *σ*^2^). In this case, conditioning on cluster *c*

(7)

where



Similar to the case of discrete traits, the likelihood function for model parameters ***β ***is given by

(8)

In this case we are interested in evaluating the difference between the mean effect of individuals with *c *copies and *r *copies. This can simply be computed as



#### Covariate Adjustment

In some instances researchers are interested in assessing the effect of CNVs after adjusting for other covariates, *Z*_1_, ..., *Z*_*K *_(usually called confounding variables). In this case, the likelihood function can be written as



where

(9)

for discrete traits, and

(10)

for quantitative traits. In both cases

(11)

### Parameter estimation

In this section we address parameter estimation for the general situation of having covariates and either discrete or quantitative traits. For brevity, let ***θ ***≡ (*β*, *γ*, *σ*) (notice that for discrete traits *σ *= 1). We consider that the weights, , are known and that they are given by the surrogate variable *X *from equation (3). Therefore, they can be used in the log-likelihood calculation, resulting in

(12)

Here *P*(*y*_*i*_| = *c*, *Z*, ***θ***) is given by equations (9) and (10) for discrete and quantitative traits, respectively. The maximum likelihood estimators (MLE) of the model parameters maximize this log-likelihood function. We propose to use a Newton-Raphson procedure to find parameter estimates. The *k*-th component of the score, *S*, is given by



The *k*-th element of the Hessian, *H*, is



where



Formulae for the derivatives of *h*_*ic *_for covariates and for discrete and qualitative traits are given in the Appendix.

MLE can be used to estimate, under the multiplicative model, the OR between individuals with copy number status *c *with respect to a reference category (e.g., individuals with copy number status *r*) as

(13)

Similarly, when analyzing continuous traits, the estimated mean effect among individuals with *c *copies with respect to those with *r *copies is

(14)

The asymptotic variance-covariance matrix of maximum likelihood estimates of ***θ ***can be estimated using the observed information matrix, *F*, as

(15)

Therefore, we can compute a 95% confidence interval (CI95%) for *OR*_*c*/*r *_using the expression

(16)

and for 

(17)

where *z*_*α*/2_ denotes the (1 - *α*/2)-th quantile of a standard normal distribution, *α *is the desired type-I error, and subindex [·, ·] denotes the position in the inverse of Fisher's information matrix.

### Hypothesis testing

We propose to use a likelihood ratio test to assess disease association, taking the model without the copy number variable as reference. Twice the increase in the log-likelihood provides the asymptotic *χ*^2 ^statistic that tests *H*_0_: *β*_1 _= *β*_2 _= ... = . In many instances, we are interested in studying the trend in effect with respect to copy number status (e.g., additive model). This can be done by generalizing equation (11) in the form

(18)

where *D *is a *I *× *M *design matrix, and *ζ *is a vector of dimension *M *having the model parameters. *M *is the total number of variables included in the model, including copy number status and confounding variables (e.g., *M *=  + *K*). For example, a trend test on copy number status without covariates *D *would have the form



and the trend hypothesis on copy number status is tested using a likelihood ratio test, comparing this model with the null model. Notice that this formulation allows us to accommodate different or common effects for each latent class. In this case, parameter estimates are obtained as shown above. Formulae for the derivatives obtained in the score and Hessian, where coefficients are not shared by each latent class, are shown in the Appendix. R language functions for the methods discussed in this paper are freely available at [25]

## Results

### Simulation study

We performed computer simulation studies to empirically examine the properties of the parameter estimators developed in the previous sections. The specific goals of these studies were: (i) to evaluate the performance of the proposed likelihood ratio trend test based on the latent class model for a number of CNV measurement distributions; (ii) to examine the effect of sample size (*I*) on the distributional properties of the estimators; (iii) to examine the bias and mean square error (MSE) of the estimators; (iv) to assess the accuracy whether of the variance and parameter estimates obtained using the observed information matrix. Simulations were performed as follows: To study (i), we simulated a binary trait using 300 cases and 300 controls. The unobserved copy number statuses (e.g. latent classes) were simulated depending on 3 different copy number status ( = 3), with the proportion of individuals in each category set as *π *= (0.5, 0.4, 0.1). The trend OR was set equal to 1.5. The observed signal intensity ratio (*X *variable) were simulated as a finite mixture of  normal distributions using different means, *η*, and variances, *σ*^2^, to assess whether the separation of clusters and their variance affects power.

To study (ii)–(iv) we simulated binary and quantitative traits. For the binary trait, simulation was performed as above but simulating various scenarios of sample size (*I*), OR and proportion of individuals with each copy number status, *π*. Again, we simulated different CNV distributions by varying *η *and *σ*^2^. For quantitative traits, we used the same simulation procedure but copy number status was simulated depending on a fixed mean trait level for the reference copy number status and a desired mean difference with respect to other copy number statuses. Next, we describe the settings for the different simulation parameters. *Sample size*: We chose the values of *I*: *I *∈ {50, 300}. Although current studies are analyzing thousands of individuals, these values were chosen to evaluate the performance of our proposed method in moderately large samples. *Copy number status*: Since we were interested in evaluating the performance of the parameter estimates, we only simulated two different copy number statuses  = {1, 2}. *Odds ratio*: To assess the impact of the strength of association between the disease and CNV, we chose two values for OR: OR ∈ {1.3, 2} in order to consider a moderate association and a strong one. *Proportion of cases with normal copy number status*: To evaluate the impact of classes with different number of individuals we set *π *∈ {(0.8, 0.2), (0.5, 0.5)}. *Finite mixture*: To asses the impact of distribution of intensity ratio, *X*, we simulated two normal distributions with the following parameters: *η *∈ {1, 1.5}, which correspond to having 2 (considered as normal copy number status) and 3 copies, respectively, and *σ *∈ {(0.15, 0.15), (0.15, 0.2), (0.2, 0.2)}. In this case, these scenarios also helped us to model different situations regarding misclassification or how latent classes were separated.

We compared three different approaches. The first (NAIVE) was based on assessing association between disease and copy number status obtained using MAP from the finite mixture model (2). That is, association was assessed using a *χ*^2 ^test from Table 1. The second is the approach that has been used predominantly to date when analyzing this kind of data and is based on assigning CNV status using pre-defined thresholds (THRES). Association is then assessed using a *χ*^2 ^test. As mentioned previously, we simulated data from two mixtures of normal distributions with means of 1 and 1.5. This is equivalent to simulating individuals with 2 and 3 copies, respectively. In this situation, it is considered that individuals with intensity (or intensity-ratio) greater than 1.33 correspond to individuals with 3 copies [10]. The third method is the one proposed in this paper, based on latent class (LC) using a *χ*^2 ^test. In order to make the results comparable, the performance of LC based on likelihood ratio trend test was compared with that of the two other methods using a *χ*^2 ^trend test (e.g. 1 degree of freedom). To evaluate bias and MSE of parameter estimates, *χ*^2 ^of association was used for all three methods.

**Figure 2:**
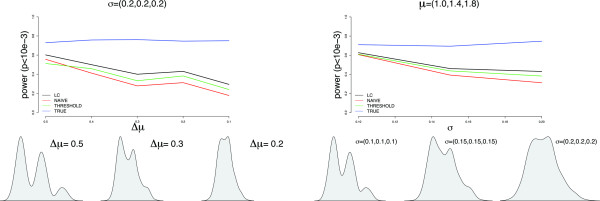
**Empirical power for simulation studies**. Empirical power for the three different approaches analyzed, varying the quality of clustering for underlying copy number status. Left panel is for fixed variance and varying means, while the right panel is for fixed mean and varying variances.

Simulation results for evaluating the performance of the likelihood ratio trend test in our proposed model are shown in Figure 2. The top figures show the power for all methods analyzed under two scenarios (other scenarios are given in Additional file 1).

The left panel shows the power for each method, varying the CNV measurement distribution with regard to the mean of each latent class, *η*, while the right panel gives the same information but with fixed means and varying variances, *σ*^2^. Figure 2 also depicts the distribution of CNV signal intensities for various scenarios. We observe that our proposed latent class model performs better in all cases, even when distribution of copy number status are not very well separated (e.g. more uncertainty).

Simulation results to evaluate parameter estimates for discrete traits are presented in Table 2 and in Table S1 and Figures S3 and S4 (see Additional file 1). Similar results and conclusions are obtained for a quantitative trait. Table 2 and Figures S3 and S4 (see Additional file 1) summarize the OR obtained by comparing individuals with 3 copies to those with 2 copies (reference category) and give the MSE for two different sample sizes, *I*, two different proportions of individuals with 2 copies, *π*, and two different variances for each component of the mixture, *σ*. Table S1 (see Additional file 1) compares different methods to compute the standard error of the ORs for the various scenarios described above. The results compare asymptotic variance based on an observed information matrix (ASYM) with respect to empirical variance (EMP). Supplementary Table S1 also shows coverage and power of confidence intervals based on the three methods analyzed. As expected, when the sample size increased, the performance of the estimators of the finite-dimensional parameters improved (Table 2). In all cases, the LC method performs better than the others. LC has less bias than NAIVE and THRES in all cases, and also shows better MSE.

**Table 2:** Simulation study

								Mean Square Error (×10^3^)		
					
I	*π*	*e*^*β*^	*σ*	SIM	NAIVE	THRES	LC	NAIVE	THRES	LC
50	0.8	1.3	(0.15,0.15)	1.23	1.17	1.15	1.20	57	87	42
50	0.8	1.3	(0.2,0.2)	1.24	1.14	1.09	1.21	107	131	114
50	0.8	1.3	(0.15,0.2)	1.28	1.18	1.15	1.24	134	148	112
50	0.8	2	(0.15,0.15)	1.60	1.40	1.28	1.48	54	85	44
50	0.8	2	(0.2,0.2)	1.82	1.36	1.29	1.52	152	158	126
50	0.8	2	(0.15,0.2)	1.89	1.42	1.33	1.57	180	253	162
50	0.5	1.3	(0.15,0.15)	1.26	1.24	1.21	1.26	39	51	32
50	0.5	1.3	(0.2,0.2)	1.32	1.28	1.25	1.35	82	79	97
50	0.5	1.3	(0.15,0.2)	1.26	1.23	1.20	1.26	66	72	60
50	0.5	2	(0.15,0.15)	2.04	1.94	1.83	2.05	40	67	34
50	0.5	2	(0.2,0.2)	2.04	1.76	1.68	2.05	107	128	92
50	0.5	2	(0.15,0.2)	2.06	1.78	1.72	1.99	87	107	71

300	0.8	1.3	(0.15,0.15)	1.30	1.25	1.18	1.30	13	32	10
300	0.8	1.3	(0.2,0.2)	1.32	1.25	1.15	1.34	27	50	29
300	0.8	1.3	(0.15,0.2)	1.30	1.22	1.16	1.29	24	42	21
300	0.8	2	(0.15,0.15)	2.01	1.87	1.49	2.01	21	120	13
300	0.8	2	(0.2,0.2)	2.03	1.70	1.36	1.99	69	203	43
300	0.8	2	(0.15,0.2)	2.03	1.62	1.38	1.86	78	189	38
300	0.5	1.3	(0.15,0.15)	1.31	1.27	1.26	1.30	7	9	5
300	0.5	1.3	(0.2,0.2)	1.30	1.23	1.22	1.30	15	17	12
300	0.5	1.3	(0.15,0.2)	1.30	1.24	1.23	1.29	12	14	9
300	0.5	2	(0.15,0.15)	2.00	1.87	1.77	2.00	11	23	5
300	0.5	2	(0.2,0.2)	2.00	1.72	1.66	2.02	36	51	15
300	0.5	2	(0.15,0.2)	2.00	1.76	1.71	1.97	26	37	10

Odds ratio (*e*^*β*^) and mean square error obtained in 1,000 simulations using the three different approaches, NAIVE, THRES and LC (see text for a description of each). Results are given for different scenarios, varying the number of individuals (*I*), the proportion of individuals with each copy number status (*π*), the odds ratio (*e*^*β*^), and the variance for CNV quantitative measurements.

Regarding variance estimates, the estimation based on ASYM showed good performance in all scenarios (see Additional file 1, Table S1). Despite slightly overestimating of EMP, the bias was less pronounced for *I *= 300, as expected. Confidence intervals based on the LC method outperform those obtained by other methods with regard to power.

### Application to real data

#### MLPA example

**Figure 3:**
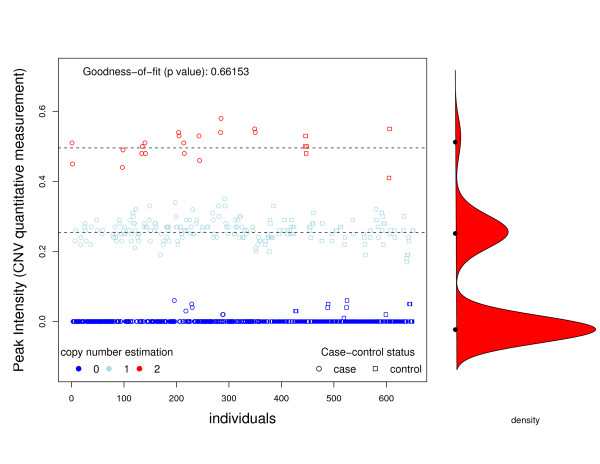
**Association between Gene 1 and disease**. Graphical representation of peak intensities (CNV quantitative measurement) of individuals for Gene 1 analyzed in the example. The various colors indicate copy number status inferred using our proposed finite mixture model.

**Figure 4:**
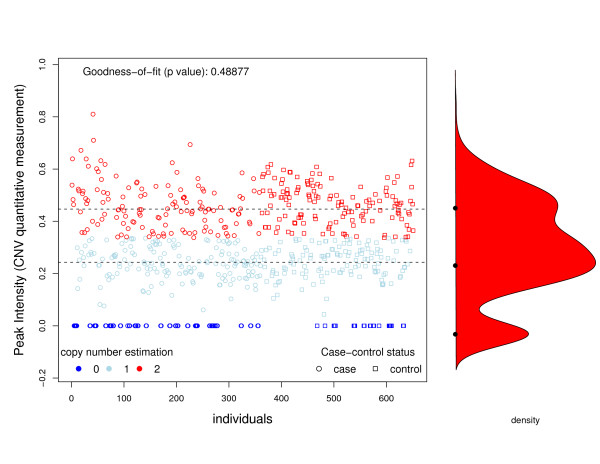
**Association between Gene 2 and disease**. Graphical representation of peak intensities (CNV quantitative measurement) of individuals for Gene 2 analyzed in the example. The various colors indicate copy number status inferred using our proposed finite mixture model.

The first data set used to analyze CNV and disease was generated and kindly provided by one of the coauthors of the current work. Although data is still unpublished, it has been made available in a blinded format for reproducing our findings using the approach presented herein, and for other validation studies. Some candidate genes were identified after performing a whole genome scan analysis using aCGH, where a pool of controls and cases were compared. In order to further investigate the relationship between the disease and altered the genes, a targeted study including several variants was designed using the MLPA technique. We obtained signal intensities of MLPA assays for 360 cases and 291 controls. Figures 3 and 4 show the intensities for cases and controls for two selected genes. In both cases, we observe 3 latent classes, corresponding to 0, 1, and 2 copies of the gene. We found that the finite mixture model fits very well (*χ*^2 ^goodness-of-fit test, *P *= 0.6615 and *P *= 0.4888). The main difference between these two cases is that copy number status for gene 1 can be established using a threshold method, while for the second gene this classification seems more arbitrary. As a consequence, misclassification should be taken into account when analyzing gene 2. Table 3 shows the classification of individuals as having 0, 1, 2 copies, estimated using equation (2) and the true copy number obtained by breakpoint cloning and assessing allele presence by PCR, which unequivocally reports the exact number of copies. From the table, we can see that the finite mixture model gives a perfect classification for gene 1 and some misclassification for gene 2. Goodness-of-fit test revealed that the proposed mixture model to determine CNV status was appropriate (p = 0.6615 and p = 0.1586).

**Table 3:** Contingency table of estimated and true copy number status for the two genes examined in the real data example.

	True copy number status		
	
	0	1	2
**Gene 1**			
0	426	0	0
1	0	201	0
2	0	0	24

**Gene 2**			
0	85	0	0
1	5	287	0
2	0	73	204

Table 4 shows the ORs and their 95%CI for the two genes analyzed. The first three columns show the results obtained in the laboratory using PCR, while the other columns show the results obtained after estimating the copy number status using our proposed finite mixture model and computing the ORs using a naïve approach (e.g. assuming that there is no misclassification) and the LC model that accounts for misclassification. As we can see, the results are the same for gene 1, since no misclassification is observed (see Figure 3 and Table 3). However, for gene 2, copy number status could not be determined as easily as for gene 1. Thus, we observe a different OR estimation and, more importantly, a different *P*-value for association. For instance, the order of magnitude of the association between the disease and gene 2 is better captured by the LC model than by the NAIVE approach. Regarding the OR estimates, the analysis using the true copy number status shows that individuals with one copy of gene 2 have a 63% decrease in disease risk with respect to individuals with 0 copies. As the 95%CI shows, this difference is statistically significant. We arrive at the same conclusion when we compare individuals with 2 copies with respect to those with 0 copies. Note that in both cases we observe that the naïve approach underestimates the OR, as shown by the simulation study.

**Table 4:** Association analysis of disease status and copy number category using the true copy number status and the estimated status obtained using the finite mixture proposed.

	True CN			Estimated CN			
	Co	Ca	OR (CI95%)	Co	Ca	OR_naïve _(CI95%)	OR_LC _(CI95%)
**Gene 1**							
0	210	216	1	210	216	1	1
1	75	126	1.63 (1.16,2.30)	75	126	1.63 (1.16,2.30)	1.63 (1.16,2.30)
2	6	18	2.92 (1.14,7.49)	6	18	2.92 (1.14,7.49)	2.92 (1.14,7.50)
*P *association			0.0027			0.0027	0.0023
*P *trend			5.0 × 10^-4^			5.0 × 10^-4^	5.0 × 10^-4^

**Gene 2**							
0	24	66	1	22	63	1	1
1	159	201	0.46 (0.27,0.77)	129	178	0.44 (0.26,0.75)	0.47 (0.27,0.82)
2	108	93	0.31 (0.18,0.54)	140	119	0.33 (0.19,0.57)	0.31 (0.18,0.54)
*P *association			7.2 × 10^-5^			2.3 × 10^-4^	8.4 × 10^-5^
*P *trend			2.1 × 10^-5^			1.0 × 10^-4^	2.1 × 10^-5^

#### aCGH example

The analysis of aCGH data requires additional steps to take into account the dependency across probes. Table 5 shows four steps we recommend for the analysis of this kind of data. First, MAP should be obtained with an algorithm that considers probe correlation. We use, in particular, the CGHcall R program which includes a mixture model to infer CNV status [18]. Second, we build blocks/regions of consecutive clones with similar signatures. To perform this step the CGHregions R library was used [26]. Third, the association between the CNV status of blocks and the trait is assessed by incorporating the uncertainty probabilities in the LC model. And fourth, corrections for multiple comparisons must be performed. We use the Benjamini-Hochberg(BH) correction [27]. This is a heuristic method that is robust against positive dependence and increasingly conservative as correlation increases [28].

**Table 5:** Steps used to assess association between CNVs and traits when aCGH is used.

**Step 1**. Use any aCGH calling procedure that provides MAP (uncertainty)
**Step 2**. Build blocks/regions of consecutive probes with similar signatures
**Step 3**. Use the signature that occurs most in a block to perform association unsing LC model
**Step 4**. Correct for multiple testing considering dependency among signatures

We applied the methodology to the breasts cancer data studied by Neve et al. [29], which is freely available from the bioconductor website [30]. The data consists on CGH arrays of 1 MB resolution [31]. The authors chose the 50 samples that could be matched to the name tokens of caArrayDB data (June 9th 2007).

In this example the association between strogen receptor positivity (dichotomous variable; 0: negative, 1: positive) and CNVs was tested. We contrasted the association as given by the LC and the NAIVE models. The original data set contained 2621 probes which were reduced to 459 blocks after the application of CGHcall and CGHregions functions. Table 6 shows the number of CNV blocks associated with strogen receptor positivity for different significance levels. We observe that incorporating classification uncertainty with the LC model substantially increased the level of association, as compared to the NAIVE approach. The number of positive association at 5% of significance after applying BH correction was 49 and 24 for LC and NAIVE approach, respectively.

**Table 6:** Number of CNV blocks (out of 459) associated with estrogen receptor positivity from 50 aCGH breast cancer cell lines.

	Significance level				
	
	10^-6^	10^-5^	10^-4^	10^-3^	10^-2^
Latent class model	1	4	27	64	117
Chi-square test	0	2	10	41	93

Results are given for different levels of association and comparing our proposed model with the naïve approach that does not consider uncertainty.

## Discussion

In this paper we have shown that the assessment of association between CNVs and disease using analysis methods that do no take into account uncertainty when inferring copy number status lead to larger p-values and underestimate the model parameters. This confounds the need to increase statistical power, which is reduced by the multiple comparison correction for the simultaneous testing of several loci. False positives are typically controlled by a dramatic reduction in the nominal p-value, such that very low values are required to reach statistical significance. Thus, a precise computation of these values is essential in genetic association studies.

Here we have proposed a latent class model (LC) that accounts for the uncertainty of assessing CNV status and also accommodates potential confounding factors. In the case of analyzing quantitative traits, we also provide formulae to further propagate call uncertainty, as other authors have proposed in another context [32]. By analyzing quantitative traits, we have assumed that the response variable follows a normal distribution, although this assumption does not hold in some instances. In this situation, one possibility is to analyze the log-transformed variable, although log transformation may not be not sufficient. The model could easily be extended to fit a response variable that has any exponential family distribution (e.g. normal, gamma, Poisson). However, we have not yet implemented this option in the functions reported here. The extension of our proposed latent-class model to assess survival time, possibly with right-censored data, is not trivial but could be a very interesting avenue for future investigation. The parameter estimation procedure proposed here, allows the estimation of confidence intervals. The LC model was remarkably consistent with simulated data. In particular, we found that the p-values obtained with the LC model were more similar to the expected values than those obtained by the threshold and naïve methods.

We maximize the likelihood function, assuming fixed weights for each copy number status, which accounts for possible misclassification. The main advantage of considering weights as known constants is that the Newton-Raphson procedure is much simpler, faster and feasible for obtaining the Hessian matrix analytically. We confirmed that the proposed model captures very well the nature of the synthetic data and variance estimates. Interestingly, we observed that the variance estimates using MLE were also reproduced when a bootstrap procedure was used (see Additional file 1, Table S2). In the interest of generalization, one can consider maximizing the likelihood function for both model parameters and weights. In that case, an EM algorithm should be used instead. However, one should bear in mind that EM does not allow for estimation of the variance of the model parameters and is computationally expensive, which may be particularly costly if this method is used in whole genome scan settings.

## Conclusion

We have shown that the LC model can incorporate uncertainty of CNV calling in the analysis. We have also illustrated how to analyze quantitative traits as well as how to accomodate confounding variables. This is of particular importance in complex diseases studies where other clinical or biochemical factors need to be taken into account. The formulation can also be generalized to assess survival times or counts in longitudinal studies. The model has showed good performance when analyzing both targeted (MLPA data) and whole genome (aCGH data) studies.

## Authors' contributions

JRG and IS developed the new statistical methods. JRG wrote the R functions and the main text of the manuscript and performed the simulation studies. GE and AC made abundant suggestions for developing the models. SP worked on the gaussian mixture approach to model quantitative CNVs measurements. XE reviewed the paper and revised its framework. LA and JRG proposed the need of a statistical tool to measure the biological differences in allele distribution in cohorts of cases and controls, and conceived the study. All authors have read, and approved the final manuscript.

## Appendix

To obtain parameter estimates, we maximize the log-likelihood function



where *P*(*y*_*i*_| = *c*, *Z*, ***θ***) is given by equations (9) and (10) for discrete and quantitative traits, respectively. As previously mentioned, the *k*-th component of the score, *S*, is given by



The *k*-th element of the Hessian, *H*, is



where



Herein we provide formulae for the derivatives of *h*_*ic *_for all cases discussed in this paper. Although the following expressions may appear complicated, they are straightforward to program and are included in the >R functions available at .

### Binary Traits

#### Binary Traits without covariates

In this case, the *h*_*ic *_function takes the form



Therefore,



where



and



#### Binary Traits with covariates

In this case, the *h*_*ic *_function takes the form



Therefore,



where



and



For covariates:



## Quantitative traits

### Quantitative traits without covariates and shared variance

In this case, the *h*_*ic *_function takes the form



Therefore,



### Quantitative traits with covariates and shared variance

In this case, the *h*_*ic *_function takes the form



Therefore,



## Trend test

In this situation we can write the linear predictor of equation (18) as



In other words, *β*_1 _plays the role of an intercept and *β*_2 _is the slope. In this case, we consider that both *β*_1 _and *beta*2 are shared for each latent class. In this situation, bearing in mind that , for the discrete traits, we have that

(19)

and

(20)

For quantitative traits, where , we have that

(21)

and

(22)

For the variance, we have that

(23)

(24)

and

(25)

## Supplementary Material

Additional file 1 Tables and figures for more scenarios of simulation studies. Click here for file

## References

[B1] Locke DP, Sharp AJ, McCarroll SA, McGrath SD, Newman TL, Cheng Z, Schwartz S, Albertson DG, Pinkel D, Altshuler DM, Eichler EE (2006). Linkage disequilibrium and heritability of copy-number polymorphisms within duplicated regions of the human genome. Am J Hum Genet.

[B2] Redon R, Ishikawa S, Fitch KR, Feuk L, Perry GH, Andrews TD, Fiegler H, Shapero MH, Carson AR, Chen W, Cho EK, Dallaire S, Freeman JL, Gonzalez JR, Grata-cos M, Huang J, Kalaitzopoulos D, Komura D, MacDonald JR, Marshall CR, Mei R, Montgomery L, Nishimura K, Okamura K, Shen F, Somerville MJ, Tchinda J, Valsesia A, Woodwark C, Yang F, Zhang J, Zerjal T, Armengol L, Conrad DF, Es-tivill X, Tyler-Smith C, Carter NP, Aburatani H, Lee C, Jones KW, Scherer SW, Hurles ME (2006). Global variation in copy number in the human genome. Nature.

[B3] Wong KK, deLeeuw RJ, Dosanjh NS, Kimm LR, Cheng Z, Horsman DE, MacAulay C, Ng RT, Brown CJ, Eichler EE, Lam WL (2007). A comprehensive analysis of common copy-number variations in the human genome. Am J Hum Genet.

[B4] Feuk L, Carson AR, Scherer SW (2006). Structural variation in the human genome. Nat Rev Genet.

[B5] Stranger BE, Forrest MS, Dunning M, Ingle CE, Beazley C, Thorne N, Redon R, Bird CP, de Grassi A, Lee C, Tyler-Smith C, Carter N, Scherer SW, Tavare S, Deloukas P, Hurles ME, Dermitzakis ET (2007). Relative impact of nucleotide and copy number variation on gene expression phenotypes. Science.

[B6] Gonzalez E, Kulkarni H, Bolivar H, Mangano A, Sanchez R, Catano G, Nibbs RJ, Freedman BI, Quinones MP, Bamshad MJ, Murthy KK, Rovin BH, Bradley W, Clark RA, Anderson SA, O'Connell RJ, Agan BK, Ahuja SS, Bologna R, Sen L, Dolan MJ, Ahuja SK (2005). The influence of CCL3L1 gene-containing segmental duplications on HIV-1/AIDS susceptibility. Science.

[B7] Rovelet-Lecrux A, Hannequin D, Raux G, Le Meur N, Laquerriere A, Vital A, Dumanchin C, Feuillette S, Brice A, Vercelletto M, Dubas F, Frebourg T, Campion D (2006). APP locus duplication causes autosomal dominant early-onset Alzheimer disease with cerebral amyloid angiopathy. Nat Genet.

[B8] Le Marechal C, Masson E, Chen JM, Morel F, Ruszniewski P, Levy P, Ferec C (2006). Hereditary pancreatitis caused by triplication of the trypsinogen locus. Nat Genet.

[B9] Schouten JP, McElgunn CJ, Waaijer R, Zwijnenburg D, Diepvens F, G P (2002). Relative quantification of 40 nucleic acid sequences by multiplex ligation-dependent probe amplification. Nucleic Acids Res.

[B10] González J, Carrasco J, Armengol L, Villatoro S, Jover L, Yasui Y, Estivill X (2008). Probe-specific mixed-model approach to detect copy number differences using multiplex ligation-dependent probe amplification (MLPA). BMC Bioinformatics.

[B11] Engert S, Wappenschmidt B, Betz B, Kast K, Kutsche M, Hellebrand H, Goecke T, Kiechle M, Niederacher D, Schmutzler R, Meindl A (2008). MLPA screening in the BRCA1 gene from 1,506 German hereditary breast cancer cases: novel deletions, frequent involvement of exon 17, and occurrence in single early-onset cases. Hum Genet.

[B12] Hansen T, Jonson L, Albrechtsen A, Andersen M, Ejlertsen B, Nielsen F (2008). Large BRCA1 and BRCA2 genomic rearrangements in Danish high risk breast-ovarian cancer families. Breast Cancer Res Treat.

[B13] Aitman T, Dong R, Vyse T, Norsworthy P, Johnson M, Smith J, Mangion J, Roberton-Lowe C, Marshall A, Petretto M, Hodges E, Bhangal G, Patel S, Sheehan-Rooney K, Duda M, Cook P, Evans D, Domin J, Flint J, Boyle J, Pusey C, Cook H (2006). Copy number polymorphism in Fcgr3 predisposes to glomerulonephritis in rats and humans. Nature.

[B14] Fellermann K, Stange D, Schaeffeler E, Schmalzl H, Wehkamp J, Bevins C, Reinisch W, Teml A, Schwab M, Lichter P, Radlwimmer B, Stange E (2006). A chromosome 8 gene-cluster polymorphism with low human beta-defensin 2 gene copy number predisposes to Crohn disease of the colon. Am J Hum Genet.

[B15] Ionita-Laza I, Rogers AJ, Lange C, Raby BA, Lee C (2009). Genetic association analysis of copy-number variation (CNV) in human disease pathogenesis. Genomics.

[B16] Fraley C, Raftery AE (1998). How many clusters? Which clustering method? Answers via model-based cluster analysis. The Computer Journal.

[B17] Picard F, Robin S, Lebarbier E, Daudin JJ (2007). A segmentation/clustering model for the analysis of array CGH data. Biometrics.

[B18] Wiel MA van de, Kim KI, Vosse SJ, van Wieringen WN, Wilting SM, Ylstra B (2007). CGHcall: calling aberrations for array CGH tumor profiles. Bioinformatics.

[B19] Leisch F (2004). A general framework for finite mixture models and latent class regression in R. Journal of Statistical Software.

[B20] Du J (2002). Combined Algorithms for Fitting Finite Mixture Distributions. PhD thesis.

[B21] Bashir S, Duffy S (1993). The correction of risk estimates for measuremente error. Ann Epidem.

[B22] Davidov O, Faraggi D, Reiser B (2003). Misclassification in logistic regression with discrete covariates. Biometrical Journal.

[B23] Greenland S (1996). Basic methods for sensitivity analysis of biases. Int J Epi.

[B24] Spiegelman D, Rosner B, Logan R (2000). Estimation and inference for logistic regression with covariate missclassification and measurement error, in main study/validation study designs. J Am Stat Assoc.

[B25] CREAL's web-page. http://www.creal.cat/jrgonzalez/software.htm.

[B26] Wiel M van de, van Wieringen W (2007). CGHregions: dimension reduction for array CGH data with minimal information loss. Cancer Informatics.

[B27] Benjamini Y, Hochberg Y (1995). Controlling the false discovery rate: A practical and powerful approach to multiple testing. J Roy Statist Soc Ser B.

[B28] Sarkar S (2006). False discovery and false nondiscovery rates in single-step multiple testing procedures. The Annals of Statistics.

[B29] Neve RM, Chin K, Fridlyand J, Yeh J, Baehner FL, Fevr T, Clark L, Bayani N, Coppe JP, Tong F, Speed T, Spellman PT, DeVries S, Lapuk A, Wang NJ, Kuo WL, Stilwell JL, Pinkel D, Albertson DG, Waldman FM, McCormick F, Dickson RB, Johnson MD, Lippman M, Ethier S, Gazdar A, Gray JW (2006). A collection of breast cancer cell lines for the study of functionally distinct cancer subtypes. Cancer Cell.

[B30] Bioconductor's web-page. http://www.bioconductor.org/.

[B31] M Neve et al in Gray Lab at LBL Neve2006: expression and CGH data on breast cancer cell lines [R package version 016].

[B32] van Wieringen WN, Wiel MA van de (2009). Nonparametric testing for DNA copy number induced differential mRNA gene expression. Biometrics.

