# Supplementary material for: Accounting for uncertainty when assessing association between copy number and disease: a latent class model
Source: BMC Bioinformatics. 2009 Jun 6;10:172. doi: 10.1186/1471-2105-10-172 (PMC2707368; doi:10.1186/1471-2105-10-172)
Supplement: Additional file 1 — Tables and figures for more scenarios of simulation studies. [file 1471-2105-10-172-S1.pdf]

Supplementary material of the paper: *Accounting for uncertainty when assessing association between copy number and disease: a latent class model.* Juan R. González, Isaac Subirana, Geòrgia Escaramís, Solymar Peraza, Alejandro Cáceres, Xavier Estivill, Lluís Armengol.

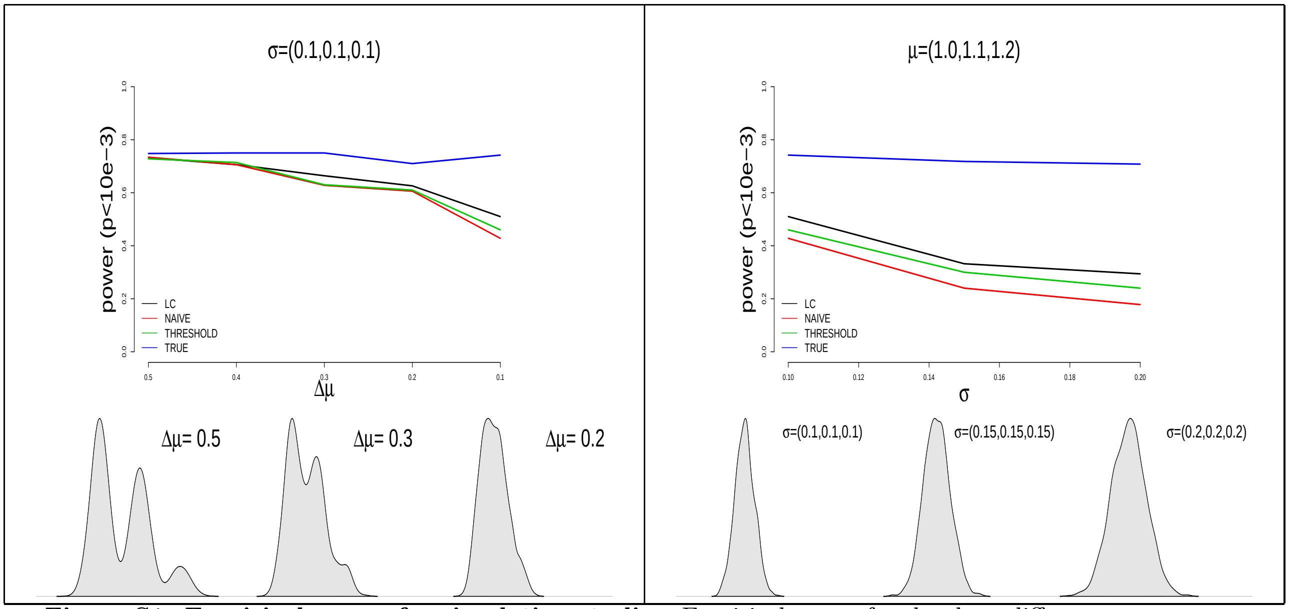

Figure S1. Empirical power for simulation studies. Empirical power for the three different approaches analyzed varying the quality of clustering for underlying copy number status. Left pannel is for a fixed set of variance and varying means, while the rigth pannel is for a fixed mean and varying variances.

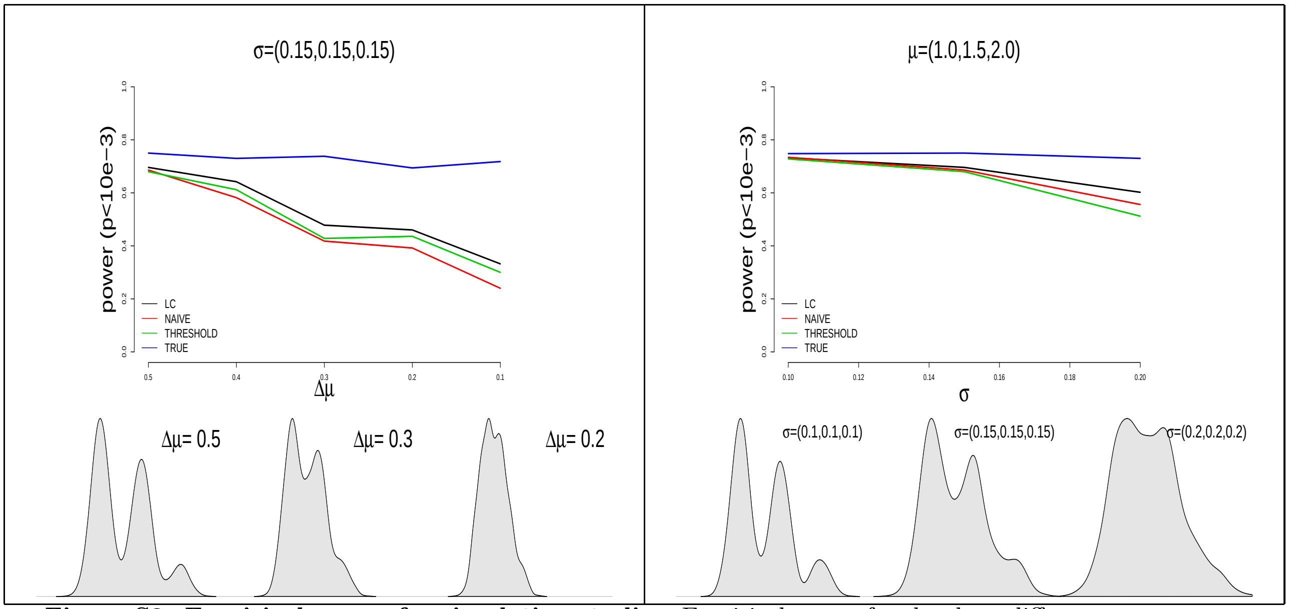

Figure S2. Empirical power for simulation studies. Empirical power for the three different approaches analyzed varying the quality of clustering for underlying copy number status. Left pannel is for a fixed set of variance and varying means, while the rigth pannel is for a fixed mean and varying variances.

		σ		
π	OR	(.15,.15)	(.2,.2)	(.15,.2)
(0.8,0.2)	1.3	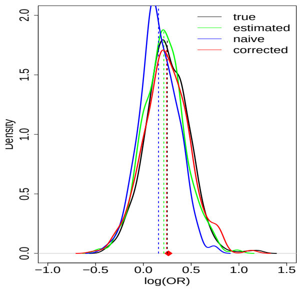	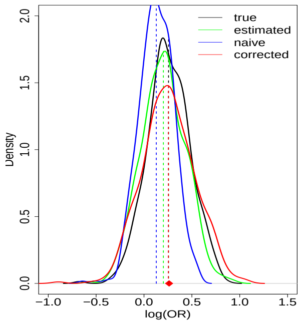	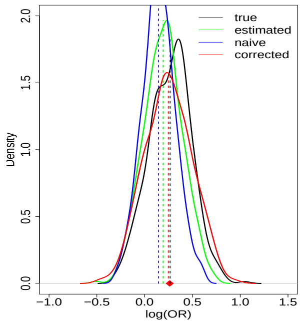
(0.5,0.5)	1.3	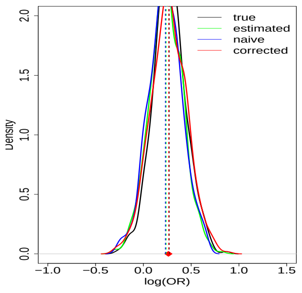	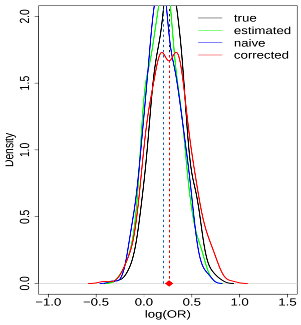	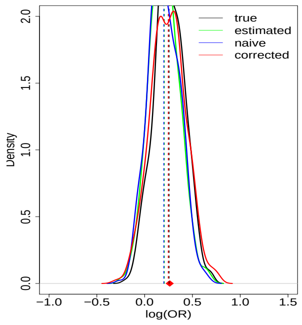
(0.8,0.2)	2	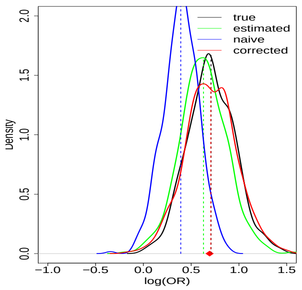	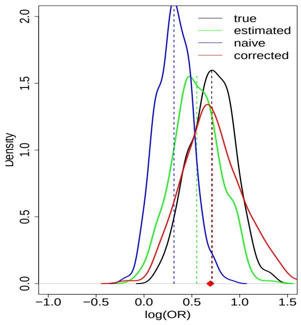	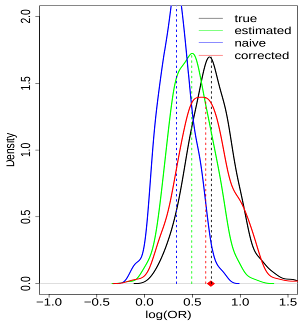
(0.5,0.5)	2	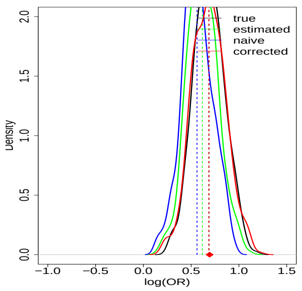	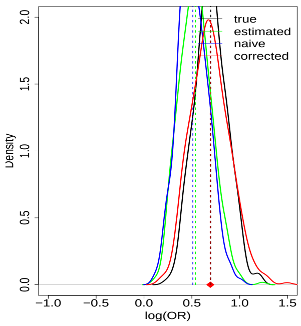	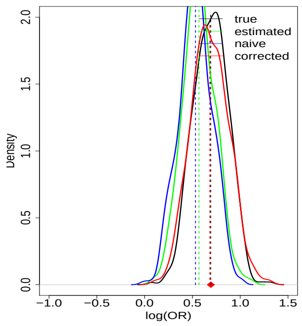

Figure S3. Empirical distribution of effect estimates (log OR) for each copy number status. Results for 1000 simulated case-control data sets (300/300), for different degrees of association (e.g. different OR) and different distributions of quantitative CNV measurements (e.g. varying clustering quality)

		σ		
π	OR	(.15,.15)	(.2,.2)	(.15,.2)
(0.8,0.2)	1.3	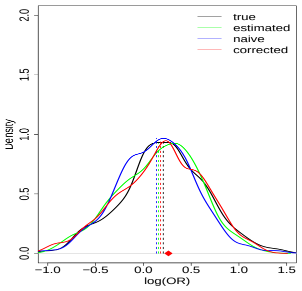	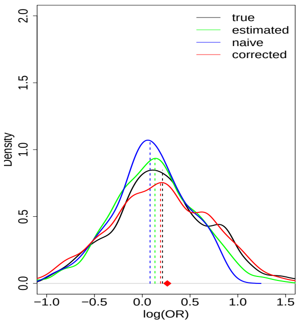	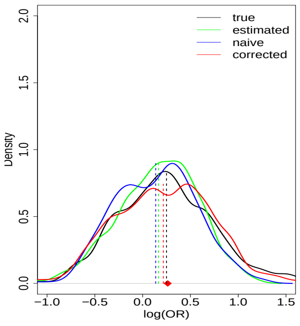
(0.5,0.5)	1.3	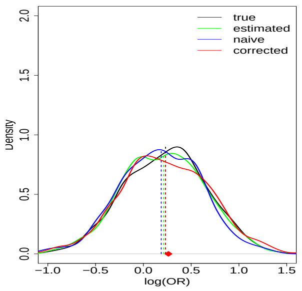	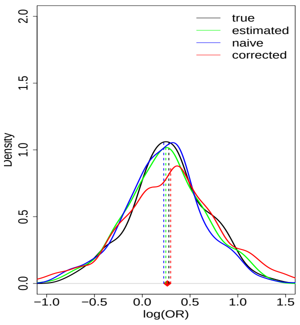	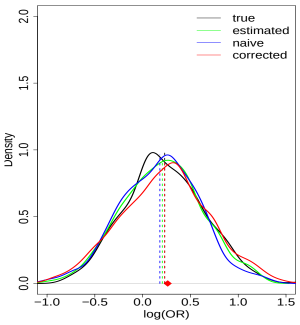
(0.8,0.2)	2	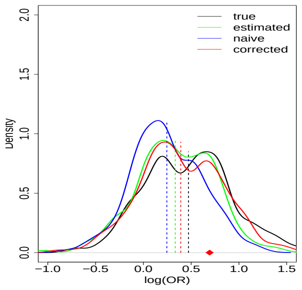	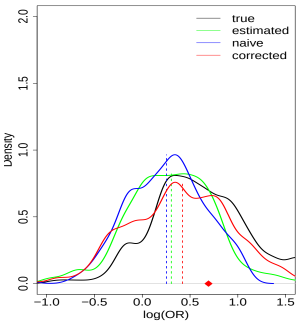	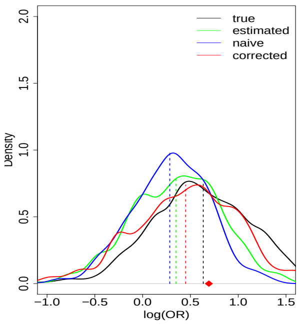
(0.5,0.5)	2	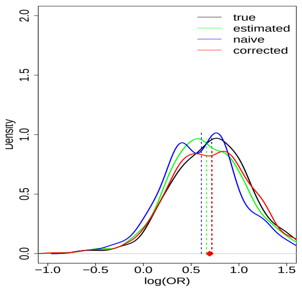	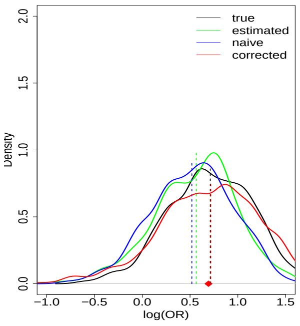	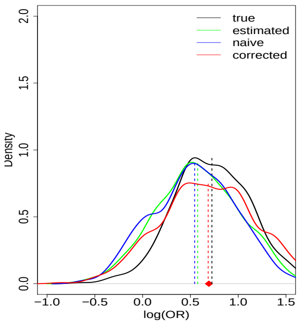

Figure S4. Empirical distribution of effect estimates (log OR) for each copy number status. Results for 1000 simulated case-control data sets (50/50), for different degrees of association (e.g. different OR) and different distributions of quantitative CNV measurements (e.g. varying clustering quality)

Table S1. Simulation study. Empirical coverage and power obtained in 1,000 simulations using the three different approaches, NAIVE, THRES and LC (see text for a description of each). Results are given for different scenarios, varying the number of individuals (I), the proportion of individuals with each copy number status (π), the odds ratio (e^{β}), and the variance for CNV quantitative measurements. The table also shows the variance of parameter estimates using the asymptotic (ASYM) variance compared with the empirical (EMP) variance.

				$\sigma_{\hat{\beta}}$		Coverage (%)				Power (%)			
I	π	e^{β}	σ	EMP	ASYM	SIM	NAIVE	THRES	LC	SIM	NAIVE	THRES	LC
50	0.8	1.3	(0.15,0.15)	0.5821	0.5898	94.2	96.2	95.8	96.8	6.6	5.4	6.4	4.6
50	0.8	1.3	(0.2,0.2)	0.5679	0.6605	93.0	94.0	93.0	96.2	5.2	4.8	4.2	3.6
50	0.8	1.3	(0.15,0.2)	0.5326	0.5846	96.6	96.2	95.4	97.4	6.8	4.8	4.8	3.0
50	0.8	2	(0.15,0.15)	0.6382	0.6512	94.2	92.6	89.0	94.0	22.0	16.8	11.2	15.4
50	0.8	2	(0.2,0.2)	0.6103	0.7057	92.8	92.2	82.8	95.2	16.8	9.4	7.4	7.0
50	0.8	2	(0.15,0.2)	0.6174	0.6407	95.6	87.0	79.4	93.0	19.4	10.6	9.8	9.6
50	0.5	1.3	(0.15,0.15)	0.4168	0.4367	94.0	94.2	95.2	93.8	11.6	10.0	9.2	10.0
50	0.5	1.3	(0.2,0.2)	0.4298	0.4838	94.6	93.8	94.0	95.4	12.6	7.0	7.0	7.2
50	0.5	1.3	(0.15,0.2)	0.3984	0.4578	95.2	95.2	95.2	95.6	11.4	8.6	9.4	8.2
50	0.5	2	(0.15,0.15)	0.4231	0.4495	95.6	94.6	93.8	94.6	39.4	32.4	32.4	32.6
50	0.5	2	(0.2,0.2)	0.4022	0.5020	97.0	95.0	94.6	98.2	42.2	23.8	23.2	25.2
50	0.5	2	(0.15,0.2)	0.4345	0.4696	94.4	93.4	94.4	95.6	47.4	30.8	29.8	33.4
300	0.8	1.3	(0.15,0.15)	0.2291	0.2341	94.0	94.0	89.2	93.2	20.4	15.2	17.0	17.8
300	0.8	1.3	(0.2,0.2)	0.2208	0.2667	94.6	94.4	88.6	96.4	23.0	17.0	11.0	16.2
300	0.8	1.3	(0.15,0.2)	0.2192	0.2373	94.2	93.6	89.2	96.0	23.4	15.8	13.2	18.0
300	0.8	2	(0.15,0.15)	0.2452	0.2610	94.2	93.6	66.0	94.6	85.4	78.4	58.6	79.2
300	0.8	2	(0.2,0.2)	0.2334	0.2996	95.8	89.8	43.2	96.0	84.2	60.8	42.6	66.6
300	0.8	2	(0.15,0.2)	0.2455	0.2591	93.8	83.0	43.8	94.6	85.8	62.8	44.8	67.4
300	0.5	1.3	(0.15,0.15)	0.1711	0.1775	93.6	93.8	94.0	93.8	37.0	30.8	31.2	32.4
300	0.5	1.3	(0.2,0.2)	0.1709	0.1970	94.4	93.8	92.8	93.6	36.6	24.4	25.0	28.2
300	0.5	1.3	(0.15,0.2)	0.1582	0.1866	96.8	95.2	94.4	95.2	34.6	22.8	24.6	25.2
300	0.5	2	(0.15,0.15)	0.1621	0.1823	95.8	95.2	90.4	95.8	98.4	96.8	93.0	97.2
300	0.5	2	(0.2,0.2)	0.1692	0.2030	96.2	84.0	82.4	96.0	99.2	89.4	90.0	94.2
300	0.5	2	(0.15,0.2)	0.1793	0.1904	95.4	88.2	83.0	95.2	98.2	92.4	88.2	94.4

Table S2. Simulation study. Empirical coverage and power obtained in 1,000 simulations using the three different approaches: NAIVE, THRES and LC (read text to have a description of each one. LCa means LC using Newton-Raphson procedure and LCb is LC using bootstrap approach). The results are given for different scenarios varying number of individuals (I), proportion of individuals in each copy number status (pi), odds ratio (e^{β}) and variance for CNV quantitative measurements. The table also shows the variance of parameter estimates using the asymptotic (ASYM) variance and variance obtained using bootstrap procedure (BOOT) compared with the empirical (EMP) variance.

				$\sigma_{\hat{\beta}}$			Coverage (%)					Power (%)				
n	π	e^{β}	σ	EMP	ASYM	BOOT	SIM	NAIVE	THRES	LCa	LCb	SIM	NAIVE	THRES	LCa	LCb
50	0.8	1.3	(0.15,0.15)	0.4539	0.5629	0.6234	66.4	66.4	65.4	66.8	66.2	1.6	1.2	1.6	1.2	1.4
50	0.8	1.3	(0.2,0.2)	0.5064	0.5985	0.6730	58.0	58.0	57.0	59.4	58.2	2.6	1.4	0.6	0.8	1.2
50	0.8	1.3	(0.15,0.2)	0.5412	0.5547	0.5991	76.2	75.8	74.6	76.6	76.2	4.2	1.8	3.6	1.6	2.4
50	0.8	2	(0.15,0.15)	0.4677	0.5886	0.6609	43.0	42.0	39.2	42.8	42.8	3.6	1.2	1.2	1.4	1.8
50	0.8	2	(0.2,0.2)	0.5105	0.6312	0.7813	46.4	43.6	39.0	45.8	45.8	6.4	2.2	3.0	2.6	4.6
50	0.8	2	(0.15,0.2)	0.5589	0.6021	0.7131	66.8	61.8	58.4	65.6	65.2	10.4	5.6	4.0	4.6	6.4
50	0.5	1.3	(0.15,0.15)	0.4357	0.4377	0.4517	94.0	93.8	94.0	94.0	93.2	10.2	9.4	7.6	7.6	8.4
50	0.5	1.3	(0.2,0.2)	0.4042	0.4864	0.5055	93.8	92.6	93.2	93.0	92.0	12.0	9.6	9.0	8.8	10.4
50	0.5	1.3	(0.15,0.2)	0.4180	0.4572	0.4765	95.0	95.2	94.6	95.6	94.4	10.2	7.6	5.6	7.4	8.8
50	0.5	2	(0.15,0.15)	0.4134	0.4500	0.4682	95.0	95.2	95.8	94.8	93.0	42.4	36.4	34.0	34.6	37.2
50	0.5	2	(0.2,0.2)	0.4461	0.5010	0.5272	91.8	89.8	90.4	91.6	90.8	42.0	29.0	27.4	30.0	33.4
50	0.5	2	(0.15,0.2)	0.4059	0.4670	0.4860	94.2	92.0	90.8	94.0	91.4	42.0	31.0	30.6	32.4	35.2
300	0.8	1.3	(0.15,0.15)	0.2167	0.2357	0.2381	95.6	96.4	91.6	96.4	95.6	23.8	18.2	14.6	19.8	23.0
300	0.8	1.3	(0.2,0.2)	0.2001	0.2680	0.2717	96.6	95.0	89.0	96.2	94.6	23.2	16.2	10.8	15.8	19.4
300	0.8	1.3	(0.15,0.2)	0.2132	0.2371	0.2400	95.8	94.4	90.2	95.0	94.0	23.2	14.6	12.8	16.6	19.2
300	0.8	2	(0.15,0.15)	0.2398	0.2592	0.2644	95.2	94.2	64.0	96.0	94.2	85.8	74.6	55.2	78.2	79.6
300	0.8	2	(0.2,0.2)	0.2469	0.2963	0.3065	93.6	87.2	38.2	95.6	94.0	86.0	56.0	39.2	63.2	66.0
300	0.8	2	(0.15,0.2)	0.2395	0.2589	0.2633	94.4	82.8	42.2	94.4	92.8	86.2	61.0	42.8	65.0	68.6
300	0.5	1.3	(0.15,0.15)	0.1580	0.1774	0.1779	95.8	96.2	94.4	95.8	94.6	35.6	28.6	27.6	29.8	32.4
300	0.5	1.3	(0.2,0.2)	0.1742	0.1967	0.1968	93.0	92.0	92.0	92.8	91.6	38.6	27.2	23.4	28.2	31.6
300	0.5	1.3	(0.15,0.2)	0.1686	0.1864	0.1878	94.0	93.8	92.4	94.6	94.2	36.6	25.6	25.2	26.6	29.2
300	0.5	2	(0.15,0.15)	0.1642	0.1825	0.1834	96.4	94.2	89.4	95.2	94.2	99.0	97.0	93.8	97.4	97.8
300	0.5	2	(0.2,0.2)	0.1681	0.2033	0.2054	95.4	86.8	80.4	94.4	92.4	98.6	90.4	88.2	94.6	94.4
300	0.5	2	(0.15,0.2)	0.1647	0.1903	0.1911	96.2	89.4	84.4	94.2	92.4	98.8	93.6	91.2	95.4	95.4
